# Optimized photo-stimulation of halorhodopsin for long-term neuronal inhibition

**DOI:** 10.1186/s12915-019-0717-6

**Published:** 2019-11-27

**Authors:** Chuanqiang Zhang, Shang Yang, Tom Flossmann, Shiqiang Gao, Otto W. Witte, Georg Nagel, Knut Holthoff, Knut Kirmse

**Affiliations:** 1 Hans-Berger Department of Neurology, Jena University Hospital, Am Klinikum 1, 07747 Jena, Germany; 2 Present Address: Laboratory of Sensory Processing, Brain Mind Institute, Faculty of Life Sciences, École Polytechnique Fédérale de Lausanne (EPFL), CH-1015 Lausanne, Switzerland; 3 Institute for Molecular Plant Physiology and Biophysics, Biocenter, & Institute of Physiology – Neurophysiology, Julius-Maximilians-University of Würzburg, 97070 Würzburg, Germany; 4 Present Address: Centre for Discovery Brain Sciences, Biomedical Sciences, University of Edinburgh, Edinburgh, EH8 9XD UK

**Keywords:** Halorhodopsin, e*Np*HR3.0, Optogenetic, Inhibition, Transgenic

## Abstract

**Background:**

Optogenetic silencing techniques have expanded the causal understanding of the functions of diverse neuronal cell types in both the healthy and diseased brain. A widely used inhibitory optogenetic actuator is e*Np*HR3.0, an improved version of the light-driven chloride pump halorhodopsin derived from *Natronomonas pharaonis*. A major drawback of e*Np*HR3.0 is related to its pronounced inactivation on a time-scale of seconds, which renders it unsuited for applications that require long-lasting silencing.

**Results:**

Using transgenic mice and *Xenopus laevis* oocytes expressing an e*Np*HR3.0-EYFP fusion protein, we here report optimized photo-stimulation techniques that profoundly increase the stability of e*Np*HR3.0-mediated currents during long-term photo-stimulation. We demonstrate that optimized photo-stimulation enables prolonged hyperpolarization and suppression of action potential discharge on a time-scale of minutes.

**Conclusions:**

Collectively, our findings extend the utility of e*Np*HR3.0 to the long-lasting inhibition of excitable cells, thus facilitating the optogenetic dissection of neural circuits.

## Background

Within a decade, optogenetic tools for reversible silencing of neurons became an integral component of the neuroscientific repertoire. They facilitate analyzing how distinct neuronal populations causally contribute to brain dynamics at the cellular, network, and behavioral level and, in addition, promise substantial therapeutic potential in diverse clinical contexts [1–3]. Optogenetic tools for neuronal inhibition are molecularly diverse, including light-activated chloride channels [4–6], potassium-specific cyclic nucleotide-gated channels fused to a photo-activated adenylyl cyclase [7, 8], G protein-coupled receptors [9, 10], and ion pumps [11–14]. All actuators developed so far have specific biophysical constraints that are of practical interest when designing and interpreting experimental studies and data, respectively [15]. For example, light-gated chloride channels (e.g., *Gt*ACR1) enable divisive inhibition by shunting excitatory currents, but the direction of ion flow entirely depends on the existing electrochemical chloride gradient and, consequently, may also depolarize rather than hyperpolarize cells [16–18]. Light-activated G protein-coupled receptors operate on slower time-scales and modulate canonical signaling cascades that, in addition to reducing excitability, could lead to undesired off-target effects, e.g., changes in gene expression [19, 20]. In contrast, light-driven ion pumps exhibit on-/off-kinetics in the millisecond range and employ subtractive inhibition, which renders them virtually independent of existing electrochemical gradients [21]. A member of the latter class of actuators is e*Np*HR3.0 [12], an improved version of the light-driven chloride pump halorhodopsin derived from *Natronomonas pharaonis* [11]. Ranking amongst the most widely used inhibitory optogenetic tools, the most critical constraint of e*Np*HR3.0 results from its prominent inactivation, which refers to a decline in photo-current amplitude during continuous illumination [11, 22–25]. Inactivation has a time constant in the range of seconds implying limited usability of e*Np*HR3.0 in experimental settings that require long-lasting (> 10 s) inhibition (for review see [15]). Based on data obtained from structurally related halorhodopsins, inactivation is thought to result from a branched photo-cycle with an accumulation of intermediates containing a deprotonated Schiff base in the 13-*cis*-retinal configuration [26]. The return to the initial state, which involves thermal reversion to all-*trans*-retinal, is slow, and few published data suggest that it may be accelerated by blue light [11, 22]. Using both transgenic mice and *Xenopus laevis* oocytes, we here systematically explore as to which extent this property could be exploited to increase the temporal stability of e*Np*HR3.0-mediated photo-currents. We provide and biophysically characterize optimized photo-stimulation protocols that greatly reduce inactivation even for prolonged illumination periods. Our findings thus extend the suitability of e*Np*HR3.0 to various experimental paradigms, including situations when long-lasting inhibition of neuronal activity is required.

## Results

### Blue light accelerates the recovery of e*Np*HR3.0-mediated currents from inactivation in a duration- and power-dependent manner

**Fig. 1:**
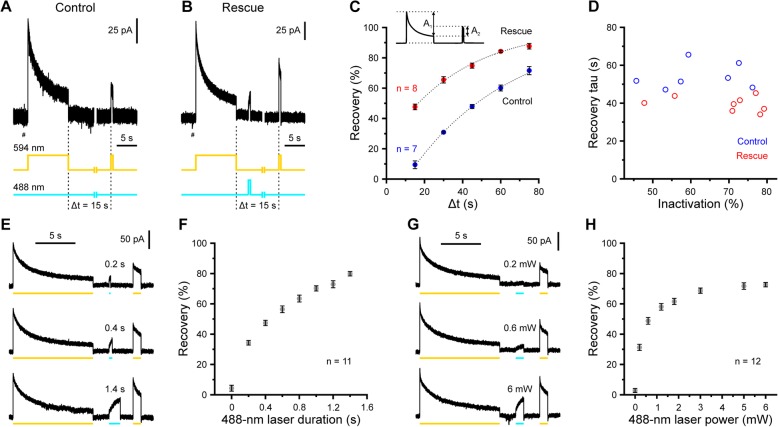
Blue light accelerates the recovery of e*Np*HR3.0-mediated currents from inactivation in a duration- and power-dependent manner. **a** Sample voltage-clamp recording illustrating that prolonged (10 s) photo-stimulation at 594 nm (5 mW) induces pronounced inactivation of e*Np*HR3.0-mediated currents. Note that the recovery from inactivation is slow (test pulse at Δ*t* = 15 s). **b** Sample trace from another cell demonstrating that blue light (500 ms, 488 nm, 5 mW) accelerates the recovery from inactivation. Also note the outward current induced by blue light. **c** Recovery of e*Np*HR3.0-mediated currents is enhanced by blue light. Inset, recovery is defined as the ratio of current amplitudes induced by the test (at Δ*t*) versus initial pulse, measured relative to *I*_late_ (i.e., recovery = *A*_2_/*A*_1_). Dotted lines represent mono-exponential fits to population data. Each cell was tested for all values of Δ*t* either without (Control, *n* = 7 cells) or with (Rescue, *n* = 8 cells) an intervening photo-stimulation at 488 nm (500 ms). In **a** and **b**, current responses to − 10-mV voltage steps used to monitor access resistance are clipped for clarity (#). **d** Independent of the degree of inactivation (1 − *I*_late_/*I*_peak_), time constants of recovery are lower for rescue as compared to control trials. Each symbol represents a single cell. **e** Recovery from inactivation depends on the duration of the 488-nm rescue pulse (blue lines). All traces are from a single cell. **f** Quantification. **g** Recovery from inactivation depends on the power of the 488-nm rescue pulse at a constant duration of 1 s. All traces are from a single cell. **h** Quantification. Data are presented as mean ± SEM

To explore the potential benefits of alternative e*Np*HR3.0 photo-stimulation paradigms, we expressed an e*Np*HR3.0-EYFP fusion protein in glutamatergic hippocampal neurons of mice using a transgenic approach (*Emx1*^*IREScre*^*:eNpHR3.0-EYFP*^*LSL*^ mice) [27]. Whole-cell voltage-clamp recordings from identified EYFP^+^ CA1 pyramidal cells were performed in the continuous presence of antagonists of voltage-gated Na^+^ channels (0.5 μM TTX) and ionotropic glutamate and GABA receptors (10 μM DNQX, 50 μM APV, 10 μM bicuculline) to abolish recurrent excitation and minimize synaptic noise. In agreement with published data, photo-stimulation using yellow light (594 nm, 5 mW at the tip of the optical fiber) induced outward currents that rapidly decayed to 34.2 ± 3.0% of the initial peak amplitude within 10 s of continuous light exposure (*I*_peak_ 62.0 ± 5.8 pA, *n* = 15 cells; Fig. 1a, b). We probed the recovery from inactivation by an additional 594-nm test pulse at variable time delays (Δ*t*) and found that it was slow under control conditions (time constant of a mono-exponential fit, 54.1 ± 2.6 s, *n* = 7 cells; Fig. 1c). Recovery from inactivation was significantly enhanced by a brief pulse of blue light (488 nm, 500 ms, 5 mW) in a Δ*t*-dependent manner [interaction (control/rescue × Δ*t*): *F* = 17.8, df = 4, *P* = 2.9 × 10^−9^, *n* = 7/8 cells (control/rescue), mixed-model ANOVA; Δ*t* = 15 s: *t*(13) = − 12.3, *P* = 1.5 × 10^−8^, Δ*t* = 30 s: *t*(8.2) = 16.1, *P* = 1.6 × 10^−7^, Δ*t* = 45 s: *t*(13) = − 12.3, *P* = 1.6 × 10^−8^, Δ*t* = 60 s: *t*(13) = 12.1, *P* = 1.9 × 10^−8^, Δ*t* = 75 s: *t*(13) = − 5.1, *P* = 2.1 × 10^−4^, two-sample *t* tests; Fig. 1b, c]. At the population level, the recovery time constant was not significantly correlated to the degree of inactivation induced by the initial photo-stimulation at 594 nm (Spearman’s rank correlation coefficient = − 0.38, *P* = 0.16, *n* = 15 cells; Fig. 1d). We next addressed the time and power requirements of blue-light rescue stimulation. In a first set of experiments, we systematically varied the duration of the 488-nm light pulse while keeping the applied power constant (5 mW). We found that the recovery from inactivation monotonically increased with increasing duration of the blue-light pulse (*F* = 575, df = 3.2, *P* = 3.4 × 10^−28^, *n* = 11 cells, one-way repeated-measures ANOVA, Huynh-Feldt correction; Fig. 1e, f). In a second set of experiments, we systematically varied the power of the 488-nm light pulse at a constant duration of 1 s. The recovery from inactivation significantly depended on the power of blue light (*F* = 529, df = 7, *P* = 2.3 × 10^−62^, *n* = 12 cells, one-way repeated-measures ANOVA; Fig. 1g, h), but saturated at close to 3 mW.

Collectively, our data demonstrate that blue light accelerates the recovery of e*Np*HR3.0-mediated currents from inactivation in a duration- and power-dependent manner.

### Blue light attenuates the inactivation of e*Np*HR3.0-mediated currents during prolonged photo-stimulation in a mean power-dependent manner

**Fig. 2:**
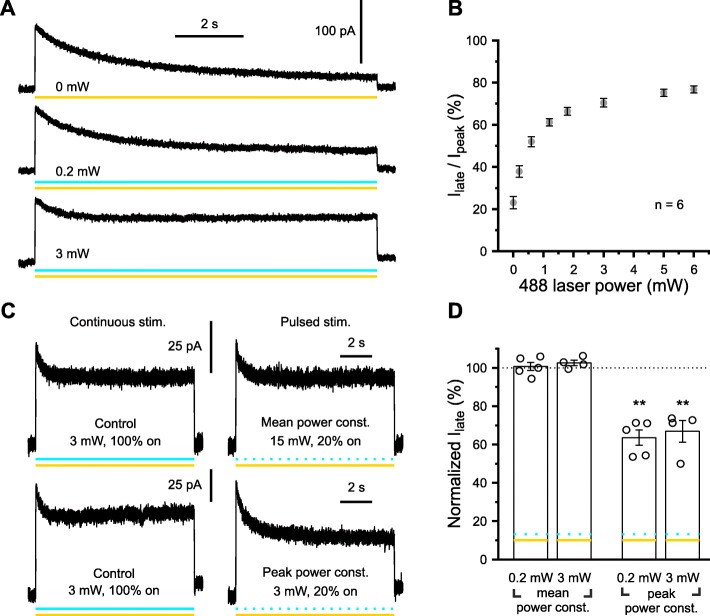
Co-stimulation at 594 nm and 488 nm attenuates the inactivation of e*Np*HR3.0-mediated currents during prolonged photo-stimulation in a mean power-dependent manner. **a** Sample voltage-clamp recording from a single cell illustrating e*Np*HR3.0-mediated currents in response to photo-stimulation at 594 nm (5 mW) alone (top) or in combination with 488 nm at variable power levels (middle and bottom). Power levels indicated refer to 488-nm light. **b** Dependence of inactivation on the power of 488-nm light. **c** The rescue effect of 488-nm light on the inactivation of e*Np*HR3.0-dependent currents depends on its mean, rather than peak, power. Top: continuous 488-nm stimulation (left) is equally effective in attenuating inactivation as compared to pulsed (1 kHz, 20/80% on/off) stimulation at constant mean power (right). Bottom*:* continuous 488-nm stimulation (left) is more effective in attenuating inactivation as compared to pulsed (1 kHz, 20/80% on/off) stimulation at constant peak power (right). **d** For quantification, *I*_late_ measured during pulsed stimulation was normalized to *I*_late_ obtained for the respective continuous-stimulation trials. Each symbol represents a single cell. Data are presented as mean ± SEM. **P < 0.01

We next assessed whether blue light may be similarly used to prevent the inactivation of e*Np*HR3.0-mediated currents when co-applied with photo-stimulation at 594 nm. To this end, we photo-stimulated cells with a constant power of 594-nm light (5 mW) and systematically varied the power of 488-nm excitation (Fig. 2a, b). We quantified this effect by determining the remaining current at the end of photo-stimulation (*I*_late_) as a fraction of the peak e*Np*HR3.0-mediated current (i.e., *I*_late_/*I*_peak_). We found that *I*_late_/*I*_peak_ strongly depended on the power of blue light (*F* = 226, df = 3.7, *P* = 3.1 × 10^−15^, *n* = 6 cells, one-way repeated-measures ANOVA, Huynh-Feldt correction; Fig. 2b) reaching an apparent saturation at about 3 mW.

We further explored the possibility to minimize the total power of blue light delivered by employing a high-frequency (“pulsed,” 1 kHz) stimulation with a 20/80% (on/off) duty cycle by means of an acousto-optic tunable filter (see the “Methods” section). In a first set of experiments, we compared the effects of continuous versus pulsed photo-stimulation at a constant mean power of either 3 mW (Fig. 2c, top) or 0.2 mW by compensatorily increasing the peak 488-nm light power in pulsed stimulation trials. At both power levels, normalized *I*_late_ amplitudes during pulsed stimulation did not significantly differ from those obtained during continuous stimulation (0.2 mW: 100.7 ± 2.1% of control, *t*(4) = − 0.63, *P* = 0.56, *n* = 5 cells, paired *t* test; 3 mW: 102.6 ± 1.4% of control, *t*(3) = − 1.98, *P* = 0.14, *n* = 4 cells, paired *t* test; Fig. 2d). In an independent set of experiments, continuous and pulsed photo-stimulation were compared at a constant peak 488-nm light power of either 3 mW (Fig. 2c, bottom) or 0.2 mW, which effectively reduced the mean power in pulsed stimulation trials to 20%. In line with the above data, pulsed stimulation at constant peak power was significantly less effective in preventing inactivation as compared to continuous excitation, reflected in lower values of *I*_late_ (0.2 mW: 63.5 ± 4.0% of control, *t*(4) = 7.11, *P* = 2.1 × 10^−3^, *n* = 5 cells, paired *t* test; 3 mW: 66.9 ± 5.7% of control, *t*(3) = 7.14, *P* = 5.7 × 10^−3^, *n* = 4 cells, paired *t* test; Fig. 2d).

Based on the same rationale and taking into consideration that the deactivation kinetics of e*Np*HR3.0-mediated currents is in the range of several milliseconds, we next investigated a potential benefit of pulsed [1 kHz, 20/80% (on/off) duty cycle] versus continuous photo-stimulation at 594 nm on the background of a continuous, constant-power (5 mW) blue-light excitation. We found that *I*_late_ amplitudes did not significantly differ between the two regimes if the mean 594-nm light power was kept constant at 3 mW by compensatorily increasing the peak power in pulsed stimulation trials (normalized *I*_late_ 96.0 ± 1.6%). In contrast, *I*_late_ was significantly reduced to 81.4 ± 1.1% if the peak 594-nm light power was unchanged (continuous/mean power = 3 mW vs. pulsed/mean power = 3 mW: *P* = 0.21, continuous/mean power = 3 mW vs. pulsed/mean power = 0.6 mW: *P* = 7.4 × 10^−4^, post hoc *t* tests with Bonferroni correction; *F* = 46.1, df = 2, *P* = 2.3 × 10^−6^, *n* = 7 cells, one-way repeated-measures ANOVA).

We further examined whether the effect of blue light reflects an inherent property of e*Np*HR3.0 by performing additional experiments on somatostatin-expressing (SOM) GABAergic interneurons (*SOM*^*IREScre*^*:eNpHR3.0-EYFP*^*LSL*^ mice) [28]. When continuously stimulated at 594 nm (5 mW), e*Np*HR3.0-mediated photo-currents decayed substantially within seconds (Additional file 1: Figure S1*A*). In contrast, alternating photo-stimulation with yellow and blue light (1 kHz, 50/50% duty cycle, each 5 mW) substantially increased *I*_late_ (*t*(4) = − 3.54, *P* = 0.024), while *I*_peak_ was moderately reduced (*t*(4) = 5.94, *P* = 4.0 × 10^−3^), resulting in a profound increase of *I*_late_/*I*_peak_ (*t*(4) = − 29.0, *P* = 8.4 × 10^−6^, *n* = 5 cells, paired *t* tests; Additional file 1: Figure S1*A–C*).

In sum, our data demonstrate that blue light attenuates the inactivation of e*Np*HR3.0-mediated currents during prolonged photo-stimulation at 594 nm in a mean power-dependent, rather than peak power-dependent, manner. Analogously, *I*_late_ is largely determined by the average, rather than the peak, 594-nm light power delivered.

### Blue light alone enables efficient and stable long-term photo-stimulation of e*Np*HR3.0

**Fig. 3:**
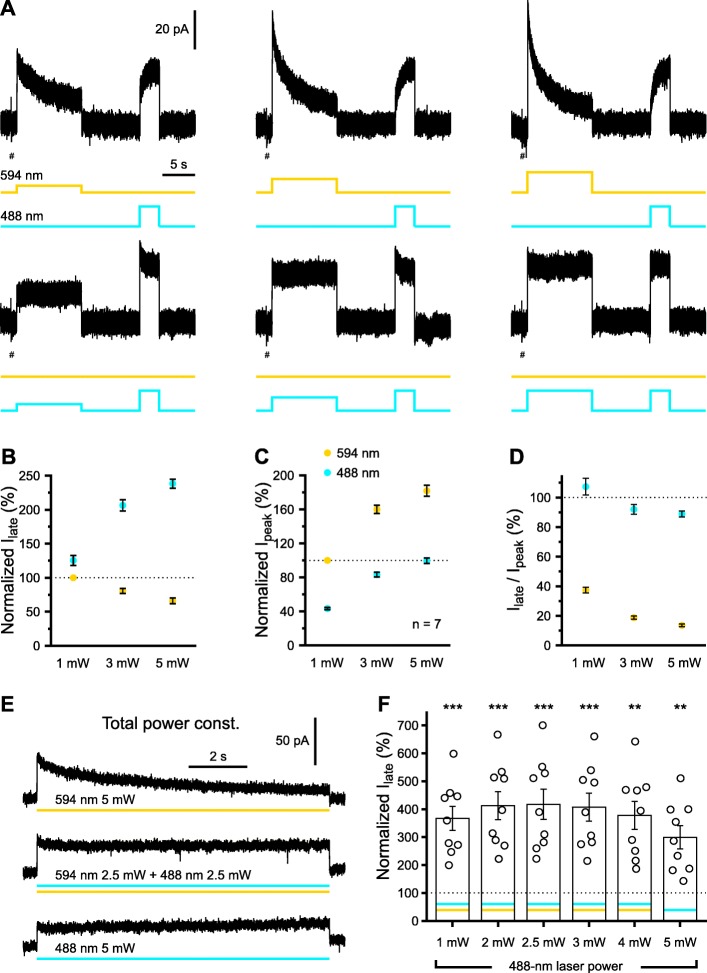
488-nm light alone enables efficient and stable long-term photo-stimulation of e*Np*HR3.0. **a** Sample voltage-clamp recordings from a single cell illustrating the power-dependence of HR-mediated currents evoked by photo-stimulation at 594 nm (top) or 488 nm (bottom), delivered at 1 mW (left), 3 mW (middle), or 5 mW (right). Note that photo-currents evoked at 488 nm display lower peak amplitudes, but high temporal stability across the entire power range examined. At the end of each trial, 488-nm light (5 mW) was used to accelerate the recovery from inactivation (note the difference in onset kinetics of evoked currents depending on the degree of previous inactivation). Current responses to − 10-mV voltage steps used to monitor access resistance are clipped for clarity (#). **b**–**d** Late (*I*_late_, **b**) and peak (*I*_peak_, **c**) current amplitudes as well as the ratio of *I*_late_ versus *I*_peak_ (**d**) normalized to the respective values at 594 nm and 1 mW (*n* = 7 cells). **e** Sample traces from a single cell photo-stimulated at 594 nm and/or 488 nm and a constant total light power of 5 mW. **f** Quantification of *I*_late_ measured during the photo-stimulation regimes indicated normalized to *I*_late_ obtained by photo-stimulation at 594 nm (5 mW) alone. Note that each combination of 594 nm plus 488 nm tested (at constant total power) considerably outperformed photo-stimulation at 594 nm alone (dotted line). Each symbol represents a single cell. Data are presented as mean ± SEM. **P < 0.01, ***P < 0.001

While the previous experiments provide a strategy to enhance the temporal stability of e*Np*HR3.0-mediated currents, our initial data employing rescue pulses of blue light (Fig. 1b) already revealed that photo-stimulation at 488 nm alone is capable of inducing outwards currents in e*Np*HR3.0-EYFP^+^ cells. We therefore set out to systematically investigate the properties of blue-light-evoked photo-stimulation of e*Np*HR3.0. To this end, cells were photo-stimulated at either 594 nm or 488 nm at power levels ranging from 1 to 5 mW (Fig. 3a). At each power level examined, *I*_peak_ values were significantly higher for yellow-light as compared to blue-light stimulation (1 mW: *t*(6) = − 6.62, *P* = 5.7 × 10^−4^; 3 mW: *t*(6) = − 7.35, *P* = 3.2 × 10^−4^; 5 mW: *t*(6) = − 6.57, *P* = 5.9 × 10^−4^; *n* = 7 cells; paired *t* tests; Fig. 3a, c). Strikingly, however, current responses evoked by blue-light photo-stimulation displayed an extraordinary temporal stability. We quantified this effect by determining *I*_late_/*I*_peak_ (Fig. 3d), which we found to be considerably higher for photo-stimulation at 488 nm as compared to 594 nm (1 mW: *t*(6) = 11.94, *P* = 2.1 × 10^−5^; 3 mW: *t*(6) = 19.69, *P* = 1.1 × 10^−6^; 5 mW: *t*(6) = 30.21, *P* = 8.7 × 10^−8^; *n* = 7 cells, paired *t* tests; Fig. 3a, d). In addition, whereas the ratio *I*_late_/*I*_peak_ strongly declined with increasing power levels for 594-nm light, this dependency was considerably weaker in case of photo-stimulation with blue light (Fig. 3a, d). As a result of this behavior, absolute *I*_late_ amplitudes evoked at 594 nm versus 488 nm diverged in a power-dependent manner [interaction (594/488 nm × power): *F* = 42.3, df = 1.06, *P* = 4.6 × 10^−4^, two-way repeated-measures ANOVA, Huynh-Feldt correction; Fig. 3b]. We next sought to determine as to which extent combinations of blue and yellow light, at constant total power (5 mW), could further increase *I*_late_. Strikingly, all tested combinations of 488/594 nm as well as 488 nm alone clearly outperformed photo-stimulation with pure yellow light as reflected in higher values of *I*_late_ (594 nm versus all other groups: *P* = 5.1 × 10^−3^ or lower, post hoc *t* tests with Bonferroni correction; *F* = 71.4, df = 1.3, *P* = 2.1 × 10^−6^, *n* = 9 cells, one-way repeated-measures ANOVA, Huynh-Feldt correction; Fig. 3e, f). The highest values of *I*_late_ were found for combinations with a blue-light fraction of 40–60%, which moderately exceeded *I*_late_ values evoked by 488 nm alone (Fig. 3f).

Collectively, our data unexpectedly reveal that photo-stimulation at 488 nm either alone or combined with 594-nm light substantially enhances non-inactivating e*Np*HR3.0-mediated currents and profoundly improves their temporal stability.

### Wavelength dependency of e*Np*HR3.0-mediated photo-currents measured in *X. laevis* oocytes

**Fig. 4:**
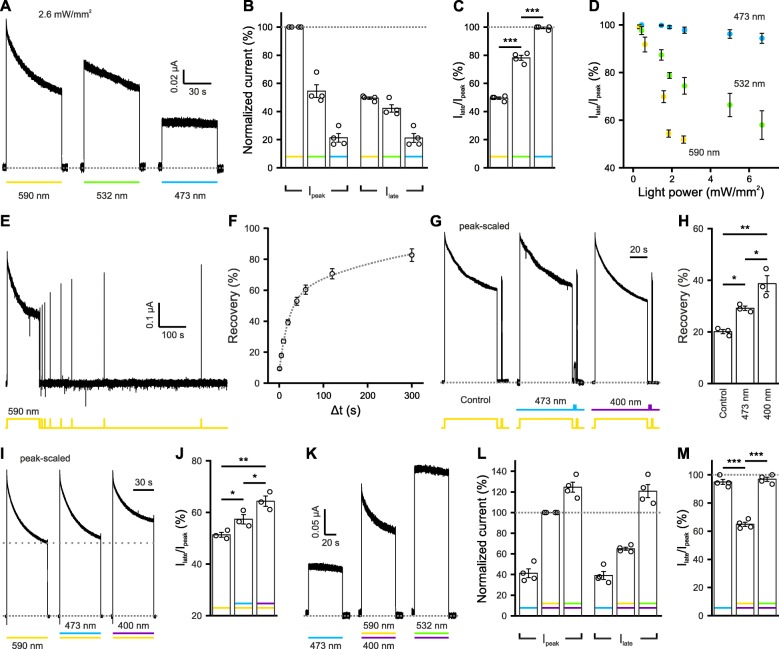
Wavelength-dependent inactivation and recovery of e*Np*HR3.0 in *X. laevis* oocytes. **a** Sample photo-current traces of e*Np*HR3.0 upon stimulation for 60 s at 590 nm, 532 nm, or 473 nm at constant intensity (2.6 mW/mm^2^). **b**, **c** Quantification of the initial peak current (*I*_peak_), the remaining current at the end of illumination (*I*_late_), and the ratio *I*_late_/*I*_peak_. **d** *I*_late_/*I*_peak_ upon 60-s-long illumination at 590 nm, 532 nm, or 473 nm at different light intensities (*n* = 5 cells). **e** Sample photo-current trace of e*Np*HR3.0. Inactivation was induced by a 60-s light pulse at 590 nm (2.6 mW/mm^2^). Recovery was probed by 10-ms light pulses (590 nm, 2.6 mW/mm^2^) at 1, 5, 10, 20, 40, 60, 120, and 300 s after the initial 60-s illumination. **f** Quantification of e*Np*HR3.0 recovery (*n* = 8 cells). **g** Peak-scaled sample traces from three different cells demonstrating that blue (473 nm) or violet (400 nm) light (2 s, 1 mW/mm^2^) accelerates the recovery from inactivation (at 5 s). Note the outward current induced by blue light. **h** Quantification of recovery as in **g**. **i** Peak-scaled sample traces from one oocyte illustrating e*Np*HR3.0 photo-currents induced by illumination at 590 nm alone (2.6 mW/mm^2^) or by co-illumination with either 473 nm (1 mW/mm^2^) or 400 nm (1 mW/mm^2^). **j** Quantification of *I*_late_/*I*_peak_ as in **i**. **k** Sample traces from one oocyte illustrating e*Np*HR3.0 photo-currents induced by illumination at 473 nm alone (6.6 mW/mm^2^), by co-illumination at 590 nm (2.6 mW/mm^2^) and 400 nm (1 mW/mm^2^) or by co-illumination at 532 nm (6.6 mW/mm^2^) and 400 nm (1 mW/mm^2^). **l**, **m** Quantification of *I*_peak_, *I*_late_, and *I*_late_/*I*_peak_ as in **k**. All measurements were performed in Ringer’s solution (pH 7.6) at a holding potential of − 40 mV. Data are presented as mean ± SEM. *P < 0.05, **P < 0.01, ***P < 0.001. **c**, **h**, **j**, **m** Asterisks indicate significance levels of post hoc *t* tests with Bonferroni correction following one-way ANOVA (**h**) or one-way repeated-measures ANOVA (**c**, **j**, **m**)

To further quantify the wavelength dependency of e*Np*HR3.0 and its generalizability to other expression systems, we next performed two-electrode voltage-clamp recordings from *X. laevis* oocytes expressing e*Np*HR3.0. We first compared e*Np*HR3.0 inactivation during long-term (60 s) illumination at three different wavelengths: 590 nm, 532 nm, and 473 nm. We found that 590-nm light could induce the highest initial photo-current amplitudes, but also showed the strongest inactivation (Fig. 4a–c) as compared to 532-nm or 473-nm light of the same intensity (2.6 mW/mm^2^; *F* = 345, df = 2, *P* = 6.4 × 10^−7^, *n* = 4 cells, one-way repeated-measures ANOVA, Fig. 4c). We next confirmed that inactivation of e*Np*HR3.0 is light power-dependent. Photo-current inactivation became more prominent with increasing power at 590 nm or 532 nm (590 nm: *F* = 194, df = 2.05, *P* = 1.1 × 10^−7^, *n* = 5 cells, one-way repeated-measures ANOVA with Greenhouse-Geisser correction; 532 nm: *F* = 21.3, df = 1.11, *P* = 0.007, *n* = 5 cells, one-way repeated-measures ANOVA with Greenhouse-Geisser correction; Fig. 4d). In agreement with our results obtained in mice, no obvious inactivation was observed for 473-nm light at powers up to 6.6 mW/mm^2^ (*F* = 3.74, df = 1.92, *P* = 0.074, *n* = 5 cells, one-way repeated-measures ANOVA with Greenhouse-Geisser correction; Fig. 4d).

Following 60-s illumination at 590 nm, e*Np*HR3.0 slowly recovered from inactivation with a weighted time constant of 200 ± 45 s in the dark (*F* = 231, df = 1.29, *P* = 5.3 × 10^−8^, *n* = 8 cells, one-way repeated-measures ANOVA, Greenhouse-Geisser correction, Fig. 4e–f). We therefore addressed whether recovery can be accelerated by short-wavelength light. While light pulses (2 s, 1 mW/mm^2^) at either 473 nm or 400 nm accelerated recovery from inactivation, violet light was found to be significantly more effective (control vs. 473 nm: *P* = 0.047; control vs. 400 nm: *P* = 1.4 × 10^−3^; 473 nm vs. 400 nm: *P* = 0.037; post hoc *t* tests with Bonferroni correction; *F* = 23.6, df = 2, *P* = 1.4 × 10^−3^, *n* = 3 cells, one-way ANOVA, Fig. 4g, h).

We next examined if short-wavelength light could alleviate e*Np*HR3.0 inactivation when co-applied with yellow light. We found that a combination of 590-nm light (2.6 mW/mm^2^) with either violet (400 nm, 1 mW/mm^2^) or blue (473 nm, 1 mW/mm^2^) light significantly increased the *I*_late_/*I*_peak_ ratio, with violet light being more effective (590 nm vs. 590 + 473 nm: *P* = 0.021; 590 nm vs. 590 + 400 nm: *P* = 1.1 × 10^− 3^; 590 + 473 nm vs. 590 + 400 nm: *P* = 0.012; post hoc *t* tests with Bonferroni correction; *F* = 61.3, df = 2, *P* = 9.9 × 10^−4^, one-way repeated-measures ANOVA, Fig. 4i, j). Violet light per se evoked very small, if any, photo-currents (*I*_400_/*I*_473_ = 6.5 ± 1.6%, *n* = 4 cells). Based on the above results, we next explored the possibility to further increase the amplitude of stable photo-currents by examining a combination of green and violet light. Here, the power of 532-nm light was set to 6.6 mW/mm^2^, which led to a similar inactivation as compared to 590-nm light at 2.6 mW/mm^2^ when applied separately (Fig. 4d). Interestingly, the combination of 1-mW/mm^2^ 400-nm light with 6.6-mW/mm^2^ 532-nm light considerably outperformed the combination with 2.6-mW/mm^2^ 590-nm light for its higher photo-current amplitudes and the eliminated inactivation (*I*_late_/*I*_peak_ for 590 + 400 nm vs. 532 + 400 nm: *P* = 2.3 × 10^−5^, post hoc *t* test with Bonferroni correction; *F* = 126, df = 2, *P* = 1.2 × 10^−5^, one-way repeated-measures ANOVA; Fig. 4k–m). In addition, whereas 6.6-mW/mm^2^ 473-nm light alone also induced stable photo-currents with negligible inactivation over 60 s (*I*_late_ vs. *I*_peak_: *t*(3) = 2.75, *P* = 0.071, *n* = 4 cells, paired *t* test; Fig. 4l), its photo-current amplitude was only about one third of the green-violet combination (Fig. 4k–m).

### Inactivation of e*Np*HR3.0 is pH- and chloride-dependent

**Fig. 5:**
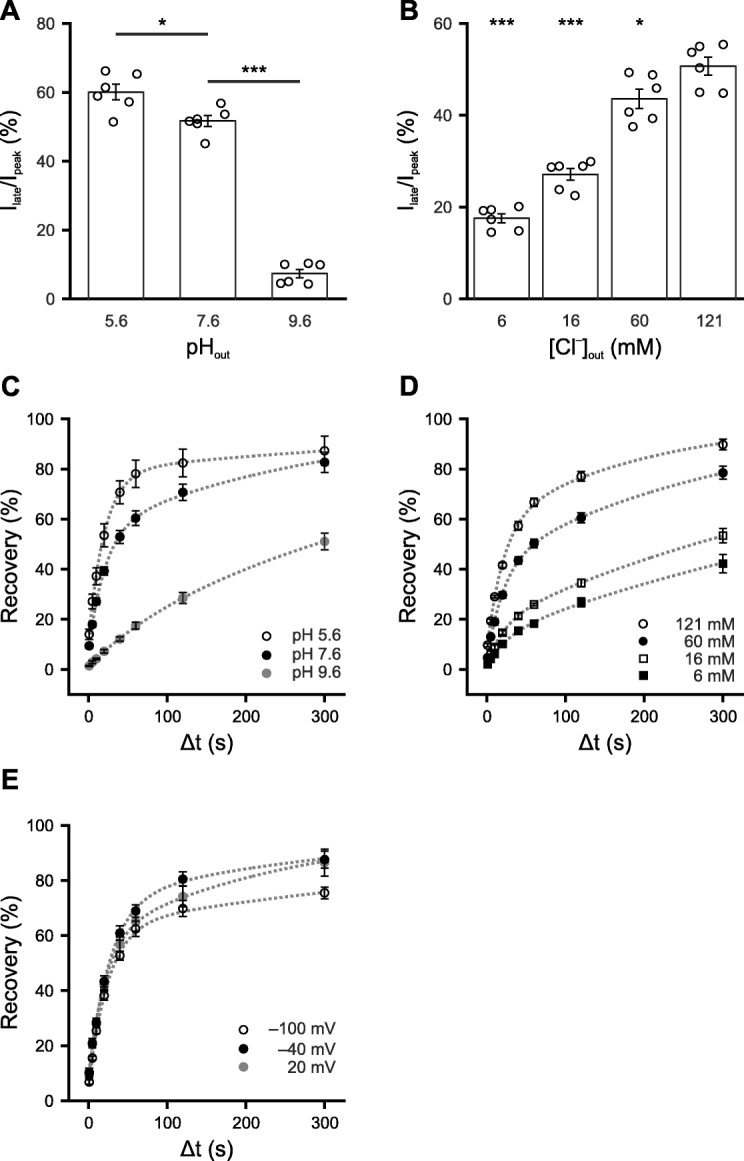
Mechanistic insight of the inactivation and recovery of e*Np*HR3.0. **a** Decreasing extracellular proton concentration enhances inactivation of e*Np*HR3.0. Currents were measured in the same oocyte in Ringer’s solution at pH 5.6, pH 7.6, or pH 9.6. **b** Increasing extracellular chloride concentration reduces inactivation of e*Np*HR3.0. Currents were measured in the same oocyte at different chloride concentrations. Buffers with different chloride concentrations were achieved by mixing Ringer’s solution (pH 7.6) and NMG-Asp solution (pH 7.6) at different ratio. **c** Recovery of e*Np*HR3.0-mediated photo-currents in Ringer’s solution at pH 5.6 (*n* = 4 cells), pH 7.6 (*n* = 8 cells), or pH 9.6 (*n* = 4 cells) at a holding potential of − 40 mV. **d** Recovery of e*Np*HR3.0-mediated photo-currents at an extracellular chloride concentration of 6 mM (*n* = 5 cells), 16 mM (*n* = 6 cells), 60 mM (*n* = 6 cells), or 121 mM (*n* = 5 cells). pH was set to 7.6 and holding potential to − 40 mV. Dotted lines in **c** and **d** represent bi-exponential fits to population data. **e** Recovery of e*Np*HR3.0 (pH 7.6) at holding potentials of − 100 mV (*n* = 7 cells), − 40 mV (*n* = 5 cells), or + 20 mV (*n* = 5 cells). Five hundred ninety-nanometer light at an intensity of 2.6 mW/mm^2^ was applied for 60 s in **a** and **b**, while in **c**–**e**, additional 10-ms 590-nm light pluses at the same intensity were delivered at 1, 5, 10, 20, 40, 60, 120, and 300 s after the initial 60-s illumination, as in Fig. 4e. Data are presented as mean ± SEM. *P < 0.05, ***P < 0.001. **a**, **b** Asterisks indicate significance levels of post hoc *t* tests with Bonferroni correction following one-way repeated-measures ANOVA

Deprotonation of the Schiff base was suggested to underlie the inactivation of *Np*HR [11, 22, 26]. We therefore investigated the effect of extracellular pH (pH_out_) on e*Np*HR3.0 inactivation. At higher pH_out_, the Schiff base is expected to lose the proton more easily. Indeed, increased inactivation was observed when pH_out_ was increased (*F* = 321, df = 2, *P* = 8.5 × 10^−10^, *n* = 6 cells, one-way repeated-measures ANOVA, Fig. 5a). Moreover, as the pK_a_ of the Schiff base is strongly dependent on the occupancy of the chloride ion at binding site I of *Np*HR [29], chloride binding to *Np*HR could stabilize the protonated Schiff base. In agreement with this, lower extracellular chloride concentration ([Cl^−^]_out_) caused stronger inactivation of e*Np*HR3.0 (*F* = 201, df = 1.4, *P* = 9.7 × 10^−7^, *n* = 6 cells, one-way repeated-measures ANOVA, Greenhouse-Geisser correction; Fig. 5b). To gain mechanistic insight into the recovery of e*Np*HR3.0 from inactivation (i.e., reprotonation of the Schiff base), we systematically investigated the effects of pH_out_, [Cl^−^]_out_ as well as membrane potential on the recovery time constant of e*Np*HR3.0 in the dark. The recovery of e*Np*HR3.0 from inactivation could be accelerated by either a decrease of pH_out_ [tests for pH_out_ effects: *F* = 61, df = 2, *P* = 2.9 × 10^−6^; interaction (pH_out_ × Δ*t*): *F* = 16.9, df = 2.66, *P* = 3.3 × 10^−5^; mixed-model ANOVA, Greenhouse-Geisser correction; Fig. 5c] or an increase of [Cl^−^]_out_ [tests for [Cl^−^]_out_ effects: *F* = 176, df = 3, *P* = 9.9 × 10^−11^; interaction ([Cl^−^]_out_ × Δ*t*): *F* = 49.6, df = 4.97, *P* = 1.5 × 10^−13^; mixed-model ANOVA, Greenhouse-Geisser correction; Fig. 5d]. This indicates that the proton for reprotonation of the Schiff base comes from the extracellular space. Interestingly, no significant difference of the recovery time of e*Np*HR3.0 at different membrane potentials was observed when [Cl^−^]_out_ was 121 mM at pH 7.6 [tests for membrane potential effects: *F* = 3.63, df = 2, *P* = 0.077; interaction (membrane potential × Δ*t*), *F* = 2.14, df = 2.85, *P* = 0.13; mixed-model ANOVA, Greenhouse-Geisser correction; Fig. 5e)]. Taken together, the data suggest that the proton for the reprotonation of the Schiff base originates from the extracellular side, and its uptake is always facilitated by the binding of chloride, and vice versa.

### Blue-light-induced photo-stimulation of e*Np*HR3.0 enables efficient long-term hyperpolarization and inhibition

**Fig. 6:**
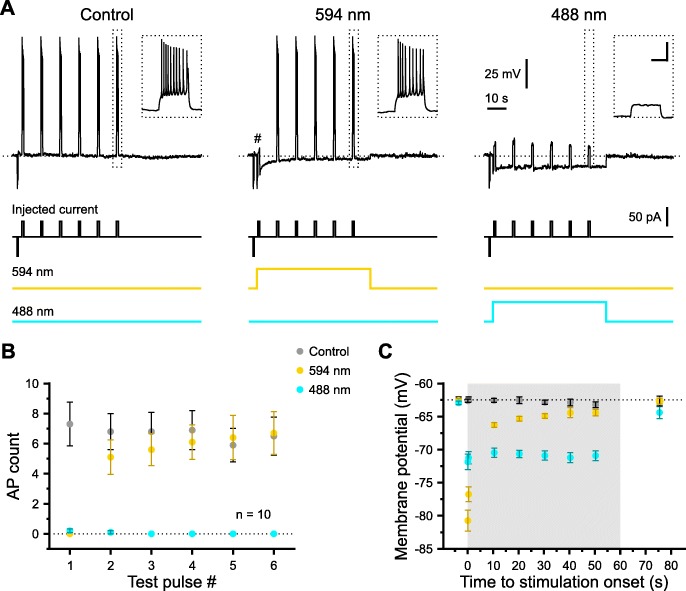
Photo-stimulation of e*Np*HR3.0 at 488 nm enables efficient long-term hyperpolarization and inhibition. **a** Sample current-clamp measurements from a single cell (biased to about − 65 mV at rest) repetitively challenged with an inward current of constant amplitude either without (5 mW, left) or with photo-stimulation at 594 nm (5 mW, middle) or 488 nm (right), respectively. Note that yellow-light stimulation initially abolished action potential firing (#), which recovered during prolonged photo-stimulation periods. Also note that blue-light stimulation suppressed firing for the entire 1-min period on the background of a stable hyperpolarization. Insets: current responses to the last test pulse at higher temporal magnification (scale bars, 25 mV, 0.5 s). **b** Number of action potentials (AP) as a function of the test-pulse number. **c** Time-course of membrane potential (gray—period of photo-stimulation). Data are presented as mean ± SEM

We next investigated whether the superior properties of photo-stimulation using short-wavelength light found in voltage-clamp experiments translate into a higher efficiency of long-term neuronal inhibition. We focused on blue light as the use of multiple wavelengths might potentially represent a complicating factor in in vivo applications (see the “Discussion” section)—in spite of lower photo-current amplitudes as compared to the green-violet combination (Fig. 4). Using current-clamp measurements from CA1 pyramidal cells in acute brain slices of mice, repetitive 1-s current injections via the patch pipette were used to evoke action potential firing in the presence of ionotropic glutamate and GABA receptor antagonists (Fig. 6a). Photo-stimulation of e*Np*HR3.0 at 594 nm (5 mW) for 1 min suppressed action potential discharge in a highly time-dependent manner: whereas firing in response to the first test pulse (500 ms after the onset of light stimulation) was virtually abolished, the inhibitory effect almost disappeared for the second as well as all subsequent test pulses (Fig. 6a, b). In contrast, photo-stimulation at 488 nm (5 mW) reliably inhibited action potential discharge during the entire 1-min illumination period [interaction (control/488 nm/594 nm × test pulse number): *F* = 13.2, df = 4.6, *P* = 1.7 × 10^−7^, *n* = 10 cells, two-way repeated-measures ANOVA, Huynh-Feldt correction; Fig. 6a, b]. Furthermore, photo-stimulation with yellow light resulted in a pronounced, but transient hyperpolarization, whereas photo-stimulation at 488 nm induced a highly stable hyperpolarizing response in all cells analyzed. Accordingly, a two-way repeated-measures ANOVA yielded a highly significant interaction between the independent variables (*F* = 71.2, df = 5.0, *P* = 3.1 × 10^−20^, *n* = 10 cells, Huynh-Feldt correction; Fig. 6a, c).

### Wavelength and duration dependence of chloride loading during photo-stimulation of e*Np*HR3.0

**Fig. 7:**
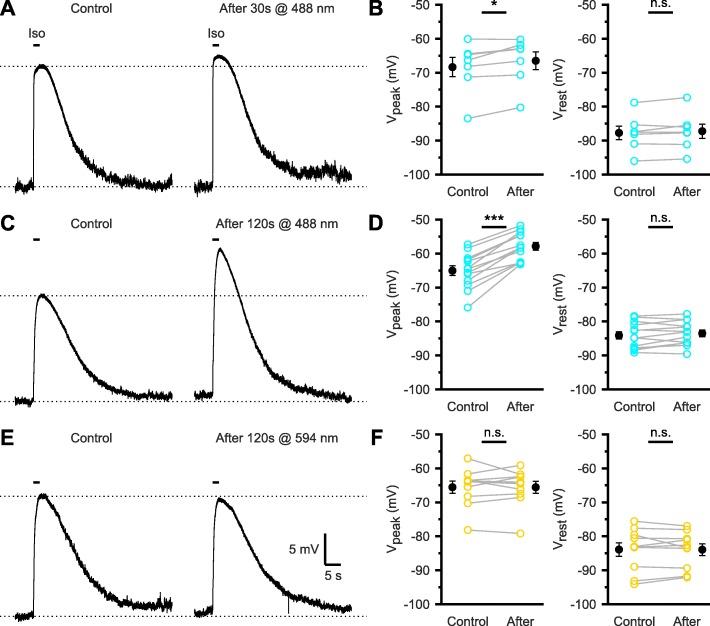
Wavelength and duration dependence of chloride loading due to photo-stimulation of e*Np*HR3.0. **a** Sample gramicidin perforated-patch recording of membrane potential in response to puff application of isoguvacine (Iso, 100 μM, 2 s) before (Control) and after photo-stimulation at 488 nm for 30 s (5 mW). Dotted lines indicate resting (*V*_rest_) and peak (*V*_peak_) membrane potential measured before photo-stimulation. Note that *V*_peak_ approximates *E*_GABA_ under our recording conditions. **b** Quantification of *V*_rest_ and *V*_peak_ before and after photo-stimulation. **c**, **d** As in **a** and **b**, but photo-stimulation was performed for 120 s at 488 nm (5 mW). **e**, **f** As in **a** and **b**, but photo-stimulation was performed for 120 s at 594 nm (5 mW). Experiments were performed at P4–10. Data are presented as mean ± SEM. n.s. not significant, **P < 0.01, ***P < 0.001

As e*Np*HR3.0 mediates chloride uptake, prolonged activation of e*Np*HR3.0 may affect [Cl^−^]_in_ and, hence, shift the reversal potential of GABA_A_ receptor-dependent currents (*E*_GABA_) into the positive direction [30]. We therefore quantified the wavelength and duration dependence of photo-stimulation-induced changes in *E*_GABA_ using gramicidin perforated-patch current-clamp recordings from CA1 pyramidal cells. In the presence of antagonists of ionotropic glutamate receptors (10 μM DNQX, 50 μM APV) and voltage-gated Na^+^ (0.5 μM TTX) and Ca^2+^ (100 μM CdCl_2_) channels, cells were challenged with a saturating puff of the GABA_A_ receptor agonist isoguvacine (100 μM, 2 s). As demonstrated before, the peak membrane potential (*V*_peak_) approximates *E*_GABA_ under these conditions [31]. We found that a 30-s-long photo-stimulation at 488 nm (5 mW) slightly, but significantly, shifted *V*_peak_ by + 1.9 ± 0.6 mV (*t*(6) = − 3.15, *P* = 0.020, *n* = 7 cells, paired *t* test; Fig. 7a, b). This effect was dose-dependent, since a more pronounced shift in *V*_peak_ by + 7.2 ± 0.7 mV (*t*(12) = − 9.77, *P* = 4.6 × 10^−7^, *n* = 13 cells, paired *t* test; Fig. 7c, d) was observed for a photo-stimulation period of 120 s (488 nm, 5 mW). In contrast, yellow-light photo-stimulation for 120 s (594 nm, 5 mW) did not significantly affect *V*_peak_ (+ 0.0 ± 0.7 mV (*t*(9) = 0.027, *P* = 0.98, *n* = 10 cells, paired *t* test; Fig. 7e, f)). Importantly, resting membrane potential (*V*_rest_) was unaffected by either photo-stimulation paradigm (*P* > 0.1, paired *t* tests; Fig. 7a–f), arguing against a major photo-toxic effect. In summary, our data demonstrate that photo-stimulation of e*Np*HR3.0 with blue light not only increases steady-state currents (Fig. 3) and membrane potential changes (Fig. 6), but also enhances the e*Np*HR3.0-mediated increase in [Cl^−^]_in_.

Consequently, alterations in network dynamics resulting from *E*_GABA_ changes represent a potential experimental constraint related to the use of e*Np*HR3.0. We examined this possibility in hippocampal slices obtained from neonatal mice (P3–6), i.e., at a developmental stage when depolarizing GABAergic transmission drives synchronized network activity [28, 32]. In agreement with published data [33], pharmacological inhibition of the chloride co-transporter NKCC1 using bumetanide (10 μM) largely abolished bursts of spontaneous postsynaptic currents (PSCs), confirming that synchronized network activity is strongly [Cl^−^]_in_-dependent at this age (Additional file 2: Figure S2*A,B*). We predicted that, in the continuous presence of bumetanide, photo-stimulation of Emx1+ CA1 pyramidal cells would rescue PSC bursts by elevating [Cl^−^]_in_. Indeed, following the offset of blue-light illumination (60 s, 5 mW), PSC bursts transiently reappeared (PSC burst count per 20-s bin: before stim 0.35 ± 0.19, after stim 4.25 ± 0.66, *t*(3) = − 6.57, *n* = 4 cells, *P* = 7.2 × 10^−3^, paired *t* test; Additional file 2: Figure S2*A–C*). Thus, these data provide proof-of-principle evidence that activation of e*Np*HR3.0 may alter neuronal population dynamics due to a shift in *E*_GABA_. We further reasoned that such effects may be less pronounced in slices obtained at a later developmental stage (P11–12), when KCC2-dependent chloride extrusion is more effective [34], synchronized network events are virtually absent and spontaneous excitatory PSCs (EPSCs) mainly reflect miniature release. In line with this prediction, long-lasting (5 min) photo-stimulation at 488 nm (5 mW) failed to alter EPSC frequency after stimulation offset, and EPSC frequency was stable for the following 10 min (*F* = 0.99, df = 2, *P* = 0.41, *n* = 6 cells, one-way repeated-measures ANOVA; Additional file 2: Figure S2*D–F*). The latter observation also argues against a major photo-toxic effect on synaptic release due to extended photo-stimulation with blue light.

## Discussion

Up to now, the chloride pump e*Np*HR3.0 is one of the most popular optogenetic tools for hyperpolarization of excitable cells. Like many other rhodopsin-based optogenetics tools, inactivation upon light illumination is a main obstacle hindering the application of e*Np*HR3.0 [24]. Here, we systematically studied the inactivation of e*Np*HR3.0 caused by long-term illumination in both murine hippocampal neurons and *X. laevis* oocytes. We expect that congruent findings made in these two different expression systems reflect the property of the protein itself, largely eliminating the influence of possible protein-protein interactions in the host system. Inactivation upon long-term illumination, slow recovery by thermal decay in the dark, and the accelerated recovery by blue-light illumination of e*Np*HR3.0 were similarly observed in both host cells. In addition, in both systems, the temporal stability of e*Np*HR3.0 is improved under optimized photo-stimulation conditions, such as co-application of yellow and blue light, green and violet light, or blue light alone.

### Biophysical mechanism of e*Np*HR3.0 inactivation and recovery

**Fig. 8:**
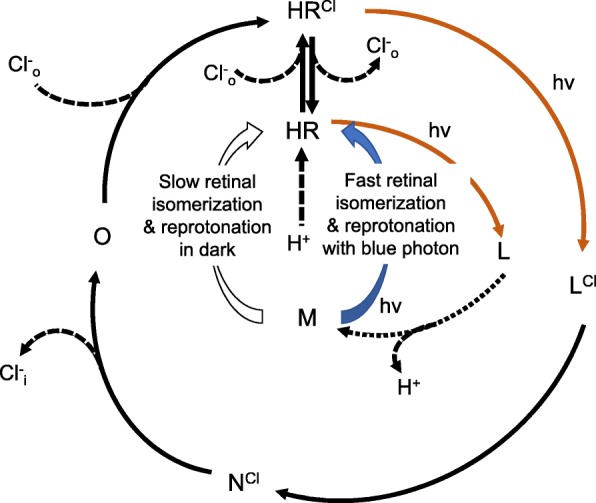
Proposed photo-cycle of *Np*HR. An extracellular chloride ion is bound to the Schiff base lysine of *Np*HR at resting state, with *K*_m_ = 16 mM [11]. Photon absorption (with maximum at 580 nm) triggers the isomerization of retinal and starts the photo-cycle, containing intermediates K (omitted here), L, N, and O. The chloride ion is released into the cytosol during the transition from N to O, and uptake of a chloride ion from the extracellular side takes place in the recovery from O to the initial state. HR without a bound chloride ion is prone to deprotonation of the Schiff base in the L state (indicated by dashed line), leading to formation of M. This intermediate is long-lived and absorbs similarly as HR_410_ (or M_412_ in BR) from *Halobacterium salinarum*. The uptake of the proton for reprotonation of the M intermediate is very slow in dark (open arrow) but fast after absorption of a blue photon (blue arrow). Our data support deprotonation of the chloride-free L state (indicated by broken line)

Inactivation of the homologue protein *Hs*HR from *Halobacterium salinarum* was proven to be the consequence of accumulation of an M-like intermediate from a branched photo-cycle with a deprotonated Schiff base [26, 35–37]. Therefore, inactivation of *Np*HR was naturally attributed to a similar mechanism, but not further investigated [11, 22]. To gain additional insights into the mechanism of inactivation of e*Np*HR3.0, we characterized the effects of different illumination and extracellular ionic conditions. Light power and wavelength dependences of inactivation may result from different photochemical processes underlying different light stimuli. The proton and chloride dependences of inactivation strongly support the Schiff base deprotonation hypothesis. Unlike bacteriorhodopsin (BR), where the positive charge of the protonated Schiff base is counterbalanced by its aspartate D85, in *Np*HR, this negative charge is provided by the binding of a chloride ion [38]. Accordingly, a decrease of either extracellular proton or chloride concentration will increase the chance of deprotonation of the Schiff base, although the Schiff base of *Np*HR was suggested to be never deprotonated during the chloride pumping cycle [39–41]. To effectively transport chloride, the Schiff base also needs to be protonated to facilitate chloride binding. Therefore, *Np*HR intermediates with deprotonated Schiff base are stable and non-pumping. Indeed, the slow kinetics of recovery from inactivation of e*Np*HR3.0 is also consistent with the stable and non-pumping feature of this M-like intermediate. Collectively, our data indicate that e*Np*HR3.0 inactivation following long-term illumination is due to formation of an M-like intermediate with a deprotonated Schiff base (Fig. 8).

We observed that the recovery time of e*Np*HR3.0 strongly depends on pH_out_, indicating that the proton for reprotonation of the deprotonatated Schiff base comes from the extracellular side. In addition, extracellular chloride could also affect the reprotonation of the Schiff base by regulating the pKa through binding to the binding site I [29]. In keeping with this, we found that lowering [Cl^−^]_out_ slowed down the recovery of e*Np*HR3.0. Changes of membrane potential will always cause opposite effects on proton or chloride binding to the Schiff base. Accordingly, no difference in recovery time was observed at different membrane potentials. It is intriguing to ask what the proton source is under the blue-light-induced recovery scenario. We argue that the proton is also from the extracellular side: First, in the structurally related *Hs*HR, proton uptake has been experimentally proven to occur from the extracellular channel upon restoration of H_410_ to the initial state by blue-light absorption [26]. Second, in BR, pump activity can be inhibited by additional blue light, in which blue-light absorption decays the M_412_ intermediate to the initial BR_568_ state by reprotonation of the Schiff base from the extracellular side [42–44] (Fig. 8). Third, a crystallography study proposed that the reprotonation of the Schiff base occurs after retinal isomerization when the cytosolic interhelical space is already closed, suggesting that the proton is from the extracellular side [45]. Beyond that, our findings may have broader utility via their application to training statistical models for the computational design of optimized *Np*HR variants [46].

### Short-wavelength light enables optimized spatiotemporal control of e*Np*HR3.0

The inactivation of *Np*HR3.0 during continuous illumination [11, 22] limits its utility for long-lasting (> 10 s) neuronal inhibition (see also [15]). However, prolonged silencing of neuronal populations is typically a critical requirement for analyzing their involvement in network oscillations and behaviors. We here confirm that inactivation increases with increasing green or yellow light power (Fig. 3), which could be particularly problematic if expression levels are low, as is the case in many transgenic models. Importantly, the present study reveals that, independent of expression system, inactivation is highly wavelength-dependent, being profoundly reduced for blue as compared to green or yellow light (Figs. 3 and 4). This may be of great practical importance as, within the tissue, light power declines with increasing distance from the fiber tip [15]. Consequently, when using yellow light, cells that lie close to the light source (i.e., that are exposed to a comparatively high light power) will not only exhibit higher peak photo-current amplitudes, but also a more pronounced to inactivation than those at larger distances. In other words, in addition to increasing the temporal stability of e*Np*HR3.0-mediated currents within individual cells, blue light is expected to minimize differences in inactivation between spatially distributed cells.

Comparing continuous with high-frequency photo-stimulation regimes revealed that blue light attenuates the yellow-light-induced e*Np*HR3.0 inactivation in a mean power-dependent manner (Fig. 2). This finding justifies the use of continuous blue-light illumination, which can be delivered using simpler hardware solutions. As compared to photo-stimulation with blue light alone, co-illumination with yellow light produced non-inactivating currents of even higher amplitude. Largest photo-currents were found for combinations with a blue-light fraction of 40–60% (Fig. 3). The temporal stability of e*Np*HR3.0-mediated currents, however, was maximal for pure 488-nm illumination and not further enhanced by co-illumination with yellow light (Fig. 3). In oocytes, similarly stable e*Np*HR3.0-mediated photo-currents were obtained by combining green with violet light, while violet light per se evoked negligible photo-currents when applied in isolation (Fig. 4). In addition, the corresponding steady-state amplitudes exceeded those evoked by photo-stimulation with pure blue light (Fig. 4). This may render the combination of green and violet light the preferred photo-stimulation regime, if high-amplitude photo-currents are required. However, it should be considered that violet and green light exhibit a differential distance-dependent power attenuation in brain tissue, which may result in larger spatial inhomogeneities as outlined above.

In sum, in combination with the fast intrinsic on-/off-kinetics of e*Np*HR3.0 in the millisecond range, the protocols described here provide for an optimized spatiotemporal control of e*Np*HR3.0 photo-activation for flexible neuronal inhibition.

### Practical considerations

Light-driven chloride pumps use subtractive inhibition (i.e., hyperpolarization) and, consequently, operate independently of the electrochemical chloride gradient [21]. This is a potential advantage over chloride-conducting channelrhodopsins (e.g., *Gt*ACR1), which act in a [Cl^−^]_in_-dependent manner and, for instance, can depolarize presynaptic terminals and evoke neurotransmitter release [18]. However, a general constraint of using ion pumps for neuronal silencing results from changes in reversal potential of the ionic species transported. For example, the proton pump archaerhodopsin (eArch3.0) was shown to induce a pH-dependent Ca^2+^ influx that enhanced spontaneous vesicular release [18]. Analogously, the chloride pump e*Np*HR3.0 can increase [Cl^−^]_in,_ which shifts *E*_GABA_ and may facilitate depolarizing GABAergic/glycinergic signaling that could counteract the e*Np*HR3.0-mediated hyperpolarization [25, 30]. We here show that *E*_GABA_ shifts are larger for photo-stimulation with blue as compared to yellow light (Fig. 7), in agreement with the increase in charge transfer for prolonged stimulation periods (Fig. 3). While manipulations of *E*_GABA_ may be useful, e.g., for dissecting the contribution of GABAergic signaling to network dynamics under physiological or pathophysiological conditions [25], they also impose an experimental constraint to the use of e*Np*HR3.0 for neuronal silencing. We provide plausibility evidence that *E*_GABA_ shifts may transiently induce aberrant network activity in the post-stimulation period at P3–6 (Additional file 2: Figure S2*A–C*), whereas such effects were not observed at a later developmental stage (Additional file 2: Figure S2*D–F*), when chloride extrusion has been shown to be more efficient [34]. In the general case, the magnitude of *E*_GABA_ shifts can hardly be predicted, as it depends on several parameters (chloride extrusion capacity, resting chloride conductance, etc.), and should be examined for a given experimental setting. Additionally, due to the fast deactivation kinetics of e*Np*HR3.0, abrupt termination of photo-stimulation can lead to rebound depolarization and action potential firing [30, 47]. This effect is due to network-based mechanisms [28], chloride loading, and/or activation of voltage-gated conductances (e.g., H-current, T-type Ca^2+^ current) and can be readily attenuated by replacing a step-like termination of photo-stimulation with a more gradual decrease in light power [18]. Finally, while our data do not provide direct evidence for photo-toxicity induced by prolonged illumination with blue light (Fig. 7 and Additional file 2: Figure S2), photo-stimulation invariably heats tissue and could thus affect a number of temperature-dependent physiological processes. Indeed, thermal constraints of optogenetics are well documented [48], further underlying the need for well-designed control experiments.

## Conclusions

Taken together, our study provides a novel approach for long-term optogenetic silencing that is based on an optimization of photo-stimulation, rather than protein engineering. For short-term optogenetic inhibition, yellow light remains the preferred choice for its capability to induce large photo-currents and its favorable tissue penetration properties. However, when prolonged inhibition is required, photo-stimulation with blue light (either alone or in combination with yellow light) is advantageous due to its superior temporal stability. Besides, our study also provides alternative photo-simulation schemes for long-term inhibition, as we observed in oocytes that a green-violet combination outperformed blue light in terms of photo-current amplitudes without any obvious inactivation. In sum, our study provides easy-to-implement photo-stimulation approaches for the light-driven chloride pump e*Np*HR3.0 that are associated with an extraordinary temporal stability of pump currents and thus render e*Np*HR3.0 suitable for long-term neuronal inhibition.

## Methods

### Ethics statement

All experimental procedures were carried out with approval from the local government and complied with European Union norms (Directive 2010/63/EU).

### Animals

Experiments were performed on acute brain slices prepared from mice of both sexes at postnatal day (P) 4–13. Pyramidal cell-specific expression of an e*Np*HR3.0-EYFP fusion protein was achieved by crossing homozygous female *Emx1*^*IREScre*^ mice (The Jackson Laboratory, stock no. 005628) [49] to homozygous male mice of the *Ai39* cre-reporting strain (The Jackson Laboratory, stock no. 014539) [50]. For experiments on SOM interneurons, homozygous *SOM*^*IREScre*^ mice (The Jackson Laboratory, stock no. 013044) [51] were crossed to homozygous *Ai39* mice. Animals were housed in standard cages with 12-h light/12-h dark cycles.

### Preparation of brain slices

Animals were decapitated under deep isoflurane anesthesia. The brain was removed quickly and transferred into ice-cold saline containing (in mM) 125 NaCl, 4 KCl, 10 glucose, 1.25 NaH_2_PO_4_, 25 NaHCO_3_, 0.5 CaCl_2_, and 2.5 MgCl_2_, bubbled with 5% CO_2_/95% O_2_ (pH, 7.4). Horizontal brain slices containing the hippocampus (350 μm) were cut on a vibratome and stored for at least 1 h before their use at room temperature in artificial cerebrospinal fluid (ACSF) containing (in mM) 125 NaCl, 4 KCl, 10 glucose, 1.25 NaH_2_PO_4_, 25 NaHCO_3_, 2 CaCl_2_, and 1 MgCl_2_, bubbled with 5% CO_2_/95% O_2_ (pH, 7.4). For recordings, slices were placed into a submerged-type recording chamber on the microscope stage (Nikon Eclipse FN1, Nikon Instruments Inc.) equipped with near-infrared differential interference contrast optics (ACSF flow rate ~ 3 ml min^−1^). Experiments were performed at near physiological temperature (32–34 °C).

### Electrophysiology in brain slices

Electrophysiological signals were acquired using a Multiclamp 700B amplifier, a 16-bit AD/DA board (Digidata 1550A) and the software pClamp 10 (Molecular Devices). Signals were low-pass filtered at 3 kHz and sampled at 10 kHz. For patch-clamp recordings of photo-currents from CA1 pyramidal cells, glass pipettes (4–7 MΩ) were filled with the following solution (in mM): 40 KCl, 100 K^+^-gluconate, 1 CaCl_2_, 11 EGTA, 10 HEPES, 2 Mg^2+^-ATP, and 0.3 Na^+^-GTP (pH adjusted to 7.25 with KOH). Whole-cell voltage-clamp recordings were performed at a holding potential of − 70 mV. In whole-cell current-clamp measurements, the resting membrane potential was manually biased to about − 65 mV via current injection. Voltages were not corrected for liquid junction potential (LJP). Except for experiments illustrated in Fig. 1a–d, a blue-light pulse (488 nm, 3 s, 5 mW) was routinely applied at the end of each stimulation trial to accelerate the recovery of e*Np*HR3.0-mediated currents from inactivation (for an example, see Fig. 3a). The recovery from inactivation of e*Np*HR3.0-mediated currents (Fig. 1c) was fitted, separately for each cell, by a mono-exponential function of the following form:

$$ \mathrm{recovery}=a\times {e}^{\frac{-\Delta  t}{\tau }}+c $$

where Δ*t* is the latency of the test pulse onset. The offset *c* was constrained to 100%.

Due to a high intercellular variability of input resistances, the amplitude of injected currents used to evoke action potential firing in current-clamp experiments (see Fig. 4) was separately set for each cell and kept constant throughout the recording. Using a series of repetitive current injections (1 s, 5-pA increments), the amplitude was determined from the largest current step that failed to induce action potentials under brief (2 s, 5 mW) photo-stimulation at 488 nm.

For gramicidin perforated-patch current-clamp recordings from CA1 pyramidal cells, glass pipettes were filled with the following solution (in mM): 140 K^+^-gluconate, 1 CaCl_2_, 11 EGTA, 1 MgCl_2_, and 10 HEPES (pH adjusted to 7.3 with KOH), additionally supplemented with 50 μg/ml gramicidin. Here, measured voltages were offline-corrected for LJP (16.5 mV). Recordings were performed at zero current.

Whole-cell voltage-clamp recordings of spontaneous PSCs were performed at a holding potential of − 70 mV without correction for LJP. At P3–6, glass pipettes were filled with (in mM) 40 KCl, 100 K^+^-gluconate, 1 CaCl_2_, 11 EGTA, 10 HEPES, 2 Mg^2+^-ATP, and 0.3 Na^+^-GTP (pH adjusted to 7.25 with KOH). Bursts of spontaneous PSCs were visually detected using the following criteria: (I) duration > 400 ms and (II) amplitude > 200 pA. For measurement of EPSCs at P11–12, glass pipettes were filled with (in mM) 8 KCl, 140 K^+^-gluconate, 1 CaCl_2_, 11 EGTA, 10 HEPES, 2 Mg^2+^-ATP, and 0.3 Na^+^-GTP (pH adjusted to 7.25 with KOH). EPSCs were detected using a template-matching algorithm implemented in pClamp 10.

### Molecular biology and genetics

e*Np*HR3.0 DNA was cloned into oocyte expression vectors, based on the plasmid pGEMHE 22, a derivative of pGEM3z (Promega). NheI-linearized plasmid DNA was used for the in vitro generation of cRNA with the AmpliCap-MaxT7 High Yield Message Maker Kit (Epicentre Biotechnologies).

### Electrophysiology in oocytes

*X. laevis* oocytes were injected with 30 ng e*Np*HR3.0 cRNA and incubated in medium containing 10 μM *all-trans*-retinal for 2 or 3 days before measurement. Two-electrode voltage-clamp recordings of photo-currents were made in Ringer’s solution with different pH (110 mM NaCl, 5 mM KCl, 2 mM CaCl_2_, 1 mM MgCl_2_, 5 mM HEPES, pH 7.6; 110 mM NaCl, 5 mM KCl, 2 mM CaCl_2_, 1 mM MgCl_2_, 5 mM MES, pH 5.6; and 110 mM NaCl, 5 mM KCl, 2 mM CaCl_2_, 1 mM MgCl_2_, 10 mM CAPSO, pH 9.6) or in NMG-Asp solution (110 mM NMG, 2 mM CaCl_2_, 1 mM MgCl_2_, 5 mM HEPES, pH adjusted to 7.6 by aspartate) at a holding potential of − 100, − 40, or 20 mV. Solutions with different chloride concentrations were prepared by mixing of Ringer’s solution with NMG-Asp solution at different ratios. The recovery from inactivation of e*Np*HR3.0-mediated currents (Fig. 4f and Fig. 5c, d) was fitted, separately for each cell, by a bi-exponential function of the following form:

$$ \mathrm{recovery}=a1\times {e}^{\frac{-\Delta  t}{\tau 1}}+a2\times {e}^{\frac{-\Delta  t}{\tau 2}}+c $$

where Δ*t* is the latency of the test pulse onset. The offset *c* was constrained to 100%. The weighted time constant *τ*_*w*_ was computed as follows:

$$ {\tau}_w=\tau 1\times \frac{a1}{a1+a2}+\tau 2\times \frac{a2}{a1+a2} $$

### Optogenetic stimulation

For measurements in brain slices, excitation was provided by a 488-nm diode laser (Cobolt MLD 488) and a 594-nm solid-state laser (Cobolt Mambo), intensity-modulated by an acousto-optic tunable filter (GH18A, Gooch & Housego) and coupled into a multimode 0.22 NA optical fiber with a core diameter of 200 μm (FG200LCC, Thorlabs GmbH). The tip of the fiber was positioned at an axial distance of ~ 0.5 mm to the surface of CA1. All power levels indicated were calibrated at the fiber tip, separately for each wavelength (LabMax-TO and OP-2 VIS sensor, Coherent). Photo-stimulation of e*Np*HR3.0 was performed using either continuous or high-frequency pulse-like (1 kHz, on/off 20/80%) stimulation patterns. The on/off time constant of the acousto-optic tunable filter was ≤ 6 μs, as quantified using a fast photodiode (PDA100A, Thorlabs).

For oocyte measurements, a 532-nm laser, a 473-nm laser (Changchun New Industries Optoelectronics Tech), a 400-nm LED (ProLight Opto Technology), and a 590-nm LED (WINGER) were used as light sources. The light intensities at different wavelengths were measured with a Laser Check optical power meter (Coherent Inc.).

### Chemicals

Chemicals were obtained from Sigma (bicuculline methiodide), Tocris [DL-2-amino-5-phosphonopentanoic acid (APV), 6,7-dinitroquinoxaline-2,3(1H,4H)-dione (DNQX)], and Biotrend [tetrodotoxin (TTX)].

### Experimental design and statistical analysis

Data were analyzed using pClamp 10, WinWCP, Microsoft Excel, and Matlab 2010a/2016a. Statistical analyses were performed using OriginPro 2018, Prism 7 and SPSS Statistics 22/24. All data are reported as mean ± standard error of the mean (SEM) (Additional file 3). Exact sample sizes for each experiment are given in the “Results” section. The Kolmogorov–Smirnov test was used to test for the normality of data. Parametric testing procedures were applied for normally distributed data; otherwise, non-parametric tests were used. In case of two-sample *t* tests and unequal group variances, Welch’s correction was applied. In case of multiple comparisons, analysis of variance (ANOVA) was used (post hoc tests indicated in the “Results” section). *P* values (two-tailed tests) < 0.05 were considered statistically significant.

## Supplementary information

**Additional file 1: Figure S1.** Co-stimulation at 594 nm and 488 nm attenuates the inactivation of e*Np*HR3.0-mediated currents in somatostatin (SOM) interneurons. *A,* Sample voltage-clamp recordings from an individual EYFP+ SOM interneuron in an acute slice obtained from a *SOM*^*IREScre*^*:eNpHR3.0-EYFP*^*LSL*^ mouse in the presence of TTX (0.5 μM). The cell was stimulated for 30 s either continuously at 594 nm (5 mW at fiber tip) or in an alternating manner at 488/594 nm (1 kHz, 50/50% duty cycle, 5 mW each at fiber tip). *B,* Quantification of I_peak_ and I_late_. *C,* Co-stimulation with blue light substantially reduced inactivation of e*Np*HR3.0-mediated photo-currents (P3–4). Data are presented as mean ± SEM. *P < 0.05, **P < 0.01, ***P < 0.001.The data set was obtained from cells included in [28].

**Additional file 2: Figure S2.** Differential effects of e*Np*HR3.0-mediated chloride loading on network activity in acute hippocampal slices. *A,* Time-course of bursts of spontaneous postsynaptic currents (PSCs) at P3–6. PSC burst were virtually absent in the presence of the NKCC1 inhibitor bumetanide (10 μM). Subsequent blue-light photo-stimulation (60 s, 488 nm, 5 mW) of Emx1+ pyramidal cells led to a transient reappearance of PSC bursts. *B,* Sample voltage-clamp recordings from an individual cell showing PSC bursts (time points as indicated in A). Note that PSC bursts reappear following photo-stimulation (bottom trace). *C,* Quantification of bursts count per 20-s time bins. *D,* Sample voltage-clamp recording of spontaneous EPSCs isolated by reversal potential before photo-stimulation (top), immediately after the offset of photo-stimulation (488 nm, 5 min, 5 mW; middle) and ~ 10 min after photo-stimulation offset (bottom). *E,* Time-course of EPSC frequency at P11–12 (normalized to the mean of the pre-stimulation period). Note the long-term stability of EPSC frequency after photo-stimulation. Brief interruptions of recordings used to monitor access resistance are not depicted for clarity *F,* Absolute EPSC frequencies before and after photo-stimulation. Data are presented as mean ± SEM. n.s. – not significant, **P < 0.01.

**Additional file 3.** Raw data values used for statistical comparisons.

## Data Availability

All data generated or analyzed during this study are included in this article and its supplementary information.
