# Supplementary figures and images for: Optimized photo-stimulation of halorhodopsin for long-term neuronal inhibition
Source: BMC Biol. 2019 Nov 27;17:95. doi: 10.1186/s12915-019-0717-6 (PMC6882325; doi:10.1186/s12915-019-0717-6)

**A***SOM*<sup>IREScree</sup>:*eNpHR3.0-EYFP*<sup>LSL</sup>

Trial#

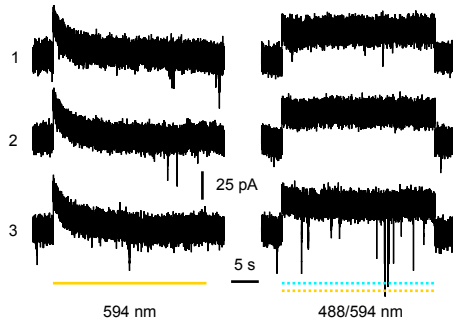**B**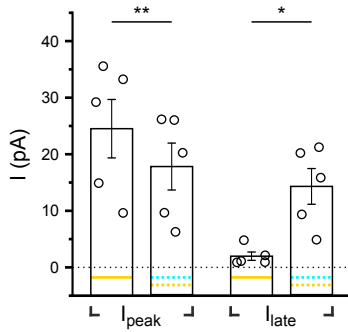**C**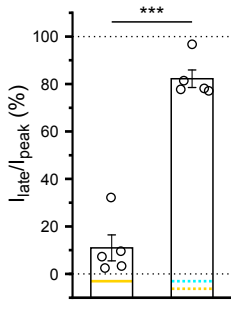

Supplement: Supplementary file 1 — Additional file 1: Figure S1. Co-stimulation at 594 nm and 488 nm attenuates the inactivation of eNpHR3.0-mediated currents in somatostatin (SOM) interneurons. A, Sample voltage-clamp recordings from an individual EYFP+ SOM interneuron in an acute slice obtained from a SOMIREScre:eNpHR3.0-EYFPLSL mouse in the presence of TTX (0.5 μM). The cell was stimulated for 30 s either continuously at 594 nm (5 mW at fiber tip) or in an alternating manner at 488/594 nm (1 kHz, 50/50% duty cycle, 5 mW each at fiber tip). B, Quantification of Ipeak and Ilate. C, Co-stimulation with blue light substantially reduced inactivation of eNpHR3.0-mediated photo-currents (P3–4). Data are presented as mean ± SEM. *P < 0.05, **P < 0.01, ***P < 0.001.The data set was obtained from cells included in [28]. [file 12915_2019_717_MOESM1_ESM.pdf]

**A** P3-6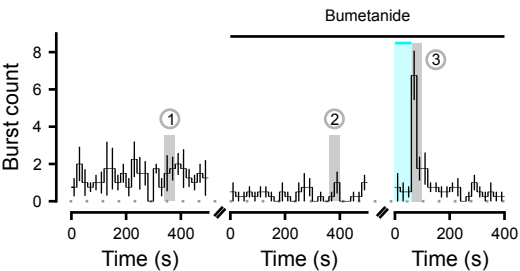**B**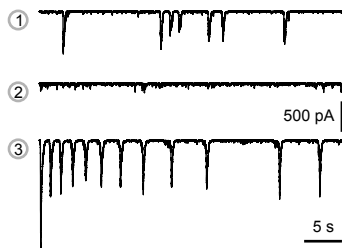**C**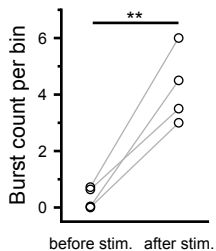**D** P11-12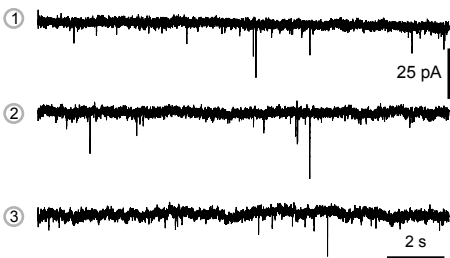**E**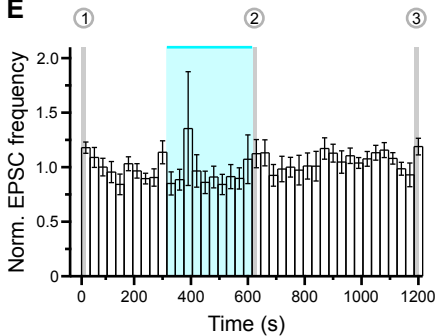**F**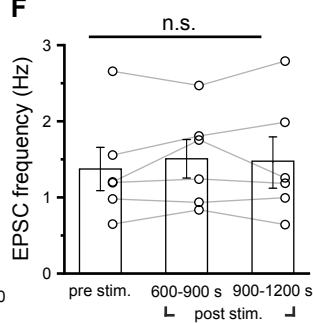

Supplement: Supplementary file 2 — Additional file 2: Figure S2. Differential effects of eNpHR3.0-mediated chloride loading on network activity in acute hippocampal slices. A, Time-course of bursts of spontaneous postsynaptic currents (PSCs) at P3–6. PSC burst were virtually absent in the presence of the NKCC1 inhibitor bumetanide (10 μM). Subsequent blue-light photo-stimulation (60 s, 488 nm, 5 mW) of Emx1+ pyramidal cells led to a transient reappearance of PSC bursts. B, Sample voltage-clamp recordings from an individual cell showing PSC bursts (time points as indicated in A). Note that PSC bursts reappear following photo-stimulation (bottom trace). C, Quantification of bursts count per 20-s time bins. D, Sample voltage-clamp recording of spontaneous EPSCs isolated by reversal potential before photo-stimulation (top), immediately after the offset of photo-stimulation (488 nm, 5 min, 5 mW; middle) and ~ 10 min after photo-stimulation offset (bottom). E, Time-course of EPSC frequency at P11–12 (normalized to the mean of the pre-stimulation period). Note the long-term stability of EPSC frequency after photo-stimulation. Brief interruptions of recordings used to monitor access resistance are not depicted for clarity F, Absolute EPSC frequencies before and after photo-stimulation. Data are presented as mean ± SEM. n.s. – not significant, **P < 0.01. [file 12915_2019_717_MOESM2_ESM.pdf]
